# Supplementary material for: End Sequence Analysis Toolkit (ESAT) expands the extractable information from single-cell RNA-seq data
Source: Genome Res. 2016 Oct;26(10):1397–410. doi: 10.1101/gr.207902.116 (PMC5052061; doi:10.1101/gr.207902.116)
Supplement: Supplemental Material [file supp_gr.207902.116_Supp_Materials.tar.gz › ESAT-esat_v0.1/doc/html/classumms_1_1core_1_1feature_1_1_gene_window.html]

ESAT: umms.core.feature.GeneWindow Class Reference


ESAT


- Main Page
- Related Pages
- Packages
- Classes
- Files

- Class List
- Class Index
- Class Hierarchy
- Class Members

All Classes Namespaces Files Functions Variables Enumerator Pages

- umms
- core
- feature
- GeneWindow

Public Member Functions |
List of all members

umms.core.feature.GeneWindow Class Reference

Inheritance diagram for umms.core.feature.GeneWindow:

[legend]

Collaboration diagram for umms.core.feature.GeneWindow:

[legend]

|  |  |
| --- | --- |
| Public Member Functions | |
|  | GeneWindow (Gene gene) |
|  | |
|  | GeneWindow (GeneWindow window) |
|  | |
|  | GeneWindow (Annotation window) |
|  | |
|  | GeneWindow (Collection<?extends Annotation > exons) |
|  | |
| int | getSize () |
|  | |
| void | addSourceAnnotation (Annotation annotation) |
|  | |
| Collection<?extends Annotation > | getSourceAnnotations () |
|  | |
| boolean | equals (Object o) |
|  | |
| int | compareTo (Annotation g) |
|  | |
| Collection< Window > | getWindows (int windowSize, int stepSize) |
|  | |
| Public Member Functions inherited from umms.core.annotation.Gene | |
|  | Gene (String chr, String name, Strand orientation, Collection<?extends Annotation > exons, int cdsStart, int cdsEnd) |
|  | |
|  | Gene (String chr, int start, int end, String name, Strand orientation, int cdsStart, int cdsEnd) |
|  | |
|  | Gene (Collection<?extends Annotation > exons, String name, Strand orientation, int cdsStart, int cdsEnd) |
|  | |
|  | Gene (String chr, String name, Strand orientation, Collection<?extends Annotation > exons) |
|  | |
|  | Gene (Annotation annotation, String name, Strand orientation, Collection<?extends Annotation > exons) |
|  | |
|  | Gene (Annotation annotation, Collection<?extends Annotation > exons) |
|  | |
|  | Gene (Annotation annotation, Collection<?extends Annotation > exons, String name, int cdsStart, int cdsEnd) |
|  | |
|  | Gene (Annotation annotation, Collection<?extends Annotation > exons, String name) |
|  | |
|  | Gene (Collection<?extends Annotation > exons, String name, Strand orientation) |
|  | |
|  | Gene (String chr, int start, int end, String name, Strand orientation) |
|  | |
|  | Gene (String chr, int start, int end, String name) |
|  | |
|  | Gene (Collection<?extends Annotation > exons, String name) |
|  | |
|  | Gene (Collection<?extends Annotation > exons) |
|  | |
|  | Gene (String chr, int start, int end) |
|  | |
|  | Gene (Alignments align) |
|  | |
|  | Gene (Annotation annotation) |
|  | |
|  | Gene (String pslString) |
|  | |
|  | Gene (String pslString, boolean isPSLFormat) |
|  | |
|  | Gene (String chr2, int start2, int end, String name2, double bedScore2, String orientation2, String sequence2, String[] extraFields2) |
|  | |
|  | Gene (String chr2, int start2, int end, String name2, double bedScore2, String orientation2, String sequence2) |
|  | |
|  | Gene (String chr, int start, int end, String name, double mybedScore, String orientation, int[] exonsStart, int[] exonsEnd) |
|  | |
|  | Gene (String chr, int start, int end, String name, String orientation, List< Integer > exonsStart, List< Integer > exonsEnd) |
|  | |
|  | Gene (String chr, int start, int end, String name, String orientation, List< Integer > exonsStart, List< Integer > exonsEnd, int blockStart, int blockEnd) |
|  | |
|  | Gene (String chr, int start, int end, String name, String orientation, List< Integer > exonsStart, List< Integer > exonsEnd, String[] extraData) |
|  | |
|  | Gene (String chr2, int start2, int end, String name2, double bedScore2, String orientation2, int[] exonStarts2, int[] exonEnds2, double[] exonScores2, String sequence2) |
|  | |
|  | Gene (String chr2, int start2, int end, String name2, double bedScore2, String orientation2, int[] exonStarts2, int[] exonEnds2, double[] exonScores2, String sequence2, String[] extraFields2) |
|  | |
|  | Gene (String chr, int start, int end, String name, double score, String strand, List< Integer > exonsStart, List< Integer > exonsEnd, int blockStart2, int blockEnd2) |
|  | |
|  | Gene (String chr, int start, int end, String name, String orientation, List< Integer > exonsStart, List< Integer > exonsEnd, int blockStart, int blockEnd, String[] extraData) |
|  | |
|  | Gene (String chr, int start, int end, String name, double score, String strand, List< Integer > exonsStart, List< Integer > exonsEnd, int blockStart2, int blockEnd2, String[] extraColumns) |
|  | |
|  | Gene (Gene gene) |
|  | |
| void | setSequence (String seq) |
|  | |
| void | setSequenceFromChromosome (Sequence chrSequence) |
|  | |
| void | setOrientation (String orientation) |
|  | |
| void | setSAMString (String sam) |
|  | |
| void | setExtraFields (double[] d) |
|  | |
| void | setExtraFields (double val, int i) |
|  | |
| void | setExtraFields (String[] extraData) |
|  | |
| int | getCDSEnd () |
|  | |
| int | getCDSStart () |
|  | |
| void | setCDSRegion (int start, int end) |
|  | |
| void | setBedScore (double d) |
|  | |
| void | setCountScore (double score) |
|  | |
| int | getSize () |
|  | |
| int | getGenomicLength () |
|  | |
| boolean | isNegativeStrand () |
|  | |
| String | getSequence () |
|  | |
| Sequence | getSequence (Sequence chrSequence) |
|  | |
| String | getSequence (Sequence chr2, boolean repeatMask, boolean stranded) throws Exception |
|  | |
| Sequence | getSequenceObject () |
|  | |
| String | getString () |
|  | |
| String | getSAMString () |
|  | |
| String | getAttribute (String name) |
|  | |
| Map | getAttributes () |
|  | |
| String[] | getExtraFields () |
|  | |
| String | getExtraFields (int i) |
|  | |
| double | getNormalizedScore () |
|  | |
| double | getRPKM () |
|  | |
| double | getPValue () |
|  | |
| double | getCountScore () |
|  | |
| double[] | getExonsScores () |
|  | |
| int | getTranscriptLength () |
|  | |
| Collection<?extends Annotation > | get5Utr () |
|  | |
| Collection<?extends Annotation > | get3Utr () |
|  | |
| Gene | get3UTRGene () |
|  | |
| Gene | get5UTRGene () |
|  | |
| Annotation | get5UTR () |
|  | |
| Annotation | get3UTR () |
|  | |
| boolean | hasCDS () |
|  | |
| Iterator< Integer > | getAllPositions () |
|  | |
| Collection< GeneWindow > | getWindows (int windowSize) |
|  | |
| Collection< GeneWindow > | getWindows (int windowSize, int stepSize, int start) |
|  | |
| Gene | getStartCodon () |
|  | |
| Annotation | get5UtrIncludingIntrons () |
|  | |
| String | getChrNum () |
|  | |
| Alignments | get3UtrIncludingIntrons () |
|  | |
| int | getNumExons () |
|  | |
| Alignments[] | getExons () |
|  | |
| Set<?extends Annotation > | getExonSet () |
|  | |
| Collection<?extends Annotation > | getSortedAndUniqueExons () |
|  | |
| IntervalTree< Annotation > | getExonTree () |
|  | |
| Annotation | getFirstExon () |
|  | |
| Annotation | getLastExon () |
|  | |
| Annotation | getOrientedLastExon () |
|  | |
| Annotation | get5PrimeExon () |
|  | |
| Annotation | get3PrimeExon (int size) |
|  | |
| Annotation | get3PrimeExon () |
|  | |
| Gene | getIntrons () |
|  | |
| Annotation | getLastIntron () |
|  | |
| Annotation | getFirstIntron () |
|  | |
| Alignments[] | getIntronsBlocks () |
|  | |
| Collection<?extends Annotation > | getIntronSet () |
|  | |
| Gene | getIntronTranscript () |
|  | |
| boolean | isCodingGene () |
|  | |
| Gene | getCDS () |
|  | |
| Alignments | getCDSRegion () |
|  | |
| Annotation | getAlignment () |
|  | |
| Alignments | getPromoter (int fudgeFactor) |
|  | |
| Alignments | getPromoter (int upstream, int downstream) |
|  | |
| double | getNumberPositions (int rate) |
|  | |
| int | getGappedSize () |
|  | |
| double | getBedScore () |
|  | |
| int | get3PrimeGenomicDistance (LightweightGenomicAnnotation annot) |
|  | |
| Gene | getOverlap (Gene second) |
|  | |
| Gene | getOverlap (Collection< Gene > others) |
|  | |
| ArrayList< Integer > | getSpliceJunctionCoordinates () throws Exception |
|  | |
| IntervalTree< Integer > | getSpliceJunctionCoordinatesTree () throws Exception |
|  | |
| String | toString () |
|  | |
| String | toRefSeq () |
|  | |
| String | toUCSC () |
|  | |
| String | toBED () |
|  | |
| String | toBED (int r, int g, int b) |
|  | |
| String | toBED (boolean useExtraFields) |
|  | |
| String | toBED (boolean useExtraFields, int r, int g, int b) |
|  | |
| String | toBEDwithBedScore () |
|  | |
| String | toGTF (String source) |
|  | |
| String | toGTF (String source, String geneName, String transcriptName) |
|  | |
| String | toCufflinksGTF (String source, String geneName, String transcriptName, String nonParsedAttrs) |
|  | |
| String | toSAM () |
|  | |
| String | toSAM (String originalSAMLine) |
|  | |
| String | toSAM (String originalSAMLine, boolean flip) |
|  | |
| void | setCDS (Annotation cdsRegion) |
|  | |
| int | absoluteToRelativePosition (int referenceCoordinate) |
|  | |
| boolean | isInExon (int snp) |
|  | |
| Collection< Gene > | findAllORFs (boolean trim3UTRs) |
|  | |
| TreeSet  < BasicLightweightAnnotation > | findAllStartCodons () |
|  | |
| Collection< Gene > | findAllORFs (Sequence chrSeq, boolean trim3UTRs) |
|  | |
| void | addSequence (Sequence seq) |
|  | |
| void | addSuffixToName (String refName) |
|  | |
| void | addAttribute (String name, String val) |
|  | |
| void | addExtraField (String value) |
|  | |
| void | expandUtrs (Integer utr1, Integer utr2) |
|  | |
| boolean | geneSpanContains (Gene gene) |
|  | |
| boolean | containsAttribute (String key) |
|  | |
| boolean | isFullyCompatible (Gene iso) |
|  | |
| boolean | overlapsExon (Annotation gen) |
|  | |
| boolean | overlapsExon (LightweightGenomicAnnotation exon) |
|  | |
| boolean | overlaps (Gene other) |
|  | |
| int | distanceToNearestNonOverlapper (Collection< Annotation > others) |
|  | |
| boolean | overlaps (Gene other, boolean ignoreOrientation) |
|  | |
| boolean | overlaps (Gene other, double minPctOverlap) |
|  | |
| boolean | overlapsStranded (Gene other, double minPctOverlap) |
|  | |
| boolean | overlapsGene (Collection< Gene > others) |
|  | |
| boolean | overlapsGene (Gene gen) |
|  | |
| boolean | overlapsGeneInAnyOrientation (Gene otherGene) |
|  | |
| boolean | overlaps2 (LightweightGenomicAnnotation next) |
|  | |
| boolean | almostEqual (Gene other, int exonFudgeFactor) |
|  | |
| boolean | almostSameStructure (Gene other, int intronFudgeFactor) |
|  | |
| boolean | almostContains (Gene other, int exonFudgeFactor) |
|  | |
| int | hashCode () |
|  | |
| int | compareTo (Annotation other) |
|  | |
| Gene | copy () |
|  | |
| GeneWindow | trimGene (int relativeStart, int relativeEnd) |
|  | |
| void | setCDSStart (int start) |
|  | |
| void | setCDSEnd (int end) |
|  | |
| Gene | findLongestORF () |
|  | |
| int | numOverlappingExons (Gene iso) |
|  | |
| int | numOfCompatibleIntrons (Gene g) |
|  | |
| GeneWindow | trimAbsolute (int alignmentStart, int alignmentEnd) |
|  | |
| double | percentOverlapping (Gene gene2) |
|  | |
| Double | percentGenomeOverlapping (Gene gene2) |
|  | |
| int | transcriptToGenomicPosition (int transcriptPosition) |
|  | |
| int | getMidpointGenomicCoords () |
|  | |
| Gene | transcriptToGenomicPosition (int startPosOnTranscript, int endPosOnTranscript) |
|  | |
| int | genomicToTranscriptPosition (int genomicPosition) |
|  | |
| int | genomicToGenomicPositionWithOffset (int genomicPosition, int offset) |
|  | |
| Gene | takeUnion (Gene other) |
|  | |
| double | getPercentCDS () |
|  | |
| double | getPercent3UTR () |
|  | |
| Gene | extendAnnotation (int deltaStart, int deltaEnd) |
|  | |
| void | addIsoform (Gene g) |
|  | |
| Collection< Gene > | getIsoforms () |
|  | |
| Public Member Functions inherited from umms.core.annotation.BasicAnnotation | |
|  | BasicAnnotation () |
|  | |
|  | BasicAnnotation (SAMRecord samRecord) |
|  | |
|  | BasicAnnotation (String ucsc) |
|  | |
|  | BasicAnnotation (String referenceName, int start, int end, Strand orientation, String name) |
|  | |
|  | BasicAnnotation (String referenceName, Strand orientation, String name, Collection<?extends Annotation > blocks) |
|  | |
|  | BasicAnnotation (String referenceName, int start, int end) |
|  | |
|  | BasicAnnotation (String referenceName, int start, int end, Strand orientation) |
|  | |
|  | BasicAnnotation (String referenceName, int start, int end, String orientation) |
|  | |
|  | BasicAnnotation (BasicAnnotation other) |
|  | |
|  | BasicAnnotation (Annotation other) |
|  | |
|  | BasicAnnotation (Collection<?extends Annotation > blocks) |
|  | |
|  | BasicAnnotation (Collection<?extends Annotation > blocks, Strand orientation, String name) |
|  | |
|  | BasicAnnotation (Collection<?extends Annotation > blocks, String name) |
|  | |
|  | BasicAnnotation (String referenceName, CompoundInterval blocks, Strand orientation, String name) |
|  | |
|  | BasicAnnotation (String referenceName, CompoundInterval blocks, Strand orientation) |
|  | |
|  | BasicAnnotation (String referenceName, CompoundInterval blocks) |
|  | |
| Annotation | copy () |
|  | |
| int | getStart () |
|  | |
| int | getEnd () |
|  | |
| int | getMidpoint () |
|  | |
| String | getReferenceName () |
|  | |
| String | getName () |
|  | |
| Strand | getOrientation () |
|  | |
| boolean | isUnoriented () |
|  | |
| List<?extends Annotation > | getBlocks () |
|  | |
| List<?extends Annotation > | getBlocks (boolean oriented) |
|  | |
| Annotation[] | getFlankingBlocks (Annotation spliceJunction) |
|  | |
| int | numBlocks () |
|  | |
| int | length () |
|  | |
| int | getReferenceCoordinateAtPosition (int positionInAnnotation, boolean ignoreOrientation) |
|  | |
| int | getPositionAtReferenceCoordinate (int referenceCoordinate, boolean ignoreOrientation) |
|  | |
| double | getScore () |
|  | |
| String | toString () |
|  | |
| void | setStart (int start) |
|  | |
| void | setEnd (int end) |
|  | |
| void | setOrientation (Strand orientation) |
|  | |
| void | setOrientedStart (int orientedStart) |
|  | |
| void | setOrientedEnd (int orientedEnd) |
|  | |
| void | setReferenceName (String refName) |
|  | |
| void | setName (String n) |
|  | |
| void | setScore (double s) |
|  | |
| void | addBlocks (Annotation block) |
|  | |
| void | addBlocks (Collection<?extends Annotation > blocks) |
|  | |
| void | shift (int delta) |
|  | |
| void | moveToCoordinate (int coordinateInReference) |
|  | |
| boolean | overlaps (Annotation other, int buffer, boolean considerOrientation) |
|  | |
| boolean | overlaps (List<?extends Annotation > otherBlocks, int buffer) |
|  | |
| int | getOverlap (Annotation other) |
|  | |
| boolean | contains (Annotation other) |
|  | |
| Annotation | union (Annotation other) |
|  | |
| Annotation | intersect (Annotation other) |
|  | |
| List< Annotation > | disect (Annotation a) |
|  | |
| List< Annotation > | disect (List<?extends Annotation > disectors) |
|  | |
| Annotation | minus (Annotation other) |
|  | |
| Annotation | minus (Collection<?extends Annotation > others) |
|  | |
| void | stitchTo (Annotation next) |
|  | |
| Annotation | complement () |
|  | |
| Collection<?extends Annotation > | getSpliceConnections () |
|  | |
| Public Member Functions inherited from umms.core.annotation.AbstractAnnotation | |
| int | size () |
|  | |
| int | getSize () |
|  | |
| int | getMidpoint () |
|  | |
| Strand | getStrand () |
|  | |
| boolean | hasOrientation () |
|  | |
| boolean | isNegativeStrand () |
|  | |
| String | getChr () |
|  | |
| int | getOrientedStart () |
|  | |
| int | getOrientedEnd () |
|  | |
| int | getLengthOnReference () |
|  | |
| int | getReferenceCoordinateAtPosition (int positionInAnnotation) |
|  | |
| int | getPositionAtReferenceCoordinate (int referenceCoordinate) |
|  | |
| void | setOrientation (char orientation) |
|  | |
| void | expand (int deltaStart, int deltaEnd) |
|  | |
| Annotation | trim (int deltaStart, int deltaEnd) |
|  | |
| boolean | fullyContains (Annotation other) |
|  | |
| boolean | overlaps (Annotation other) |
|  | |
| boolean | overlaps (Annotation other, int buffer) |
|  | |
| boolean | overlapsStranded (Annotation other) |
|  | |
| boolean | overlaps (Annotation other, boolean considerOrientation) |
|  | |
| boolean | overlaps (Collection<?extends Annotation > others) |
|  | |
| boolean | overlaps (Collection<?extends Annotation > others, int buffer) |
|  | |
| String | toUCSC () |
|  | |
| String | toBED () |
|  | |
| final String | getFullInfoString () |
|  | |
| String | toBED (int r, int g, int b) |
|  | |
| String | toShortBED () |
|  | |
| String | toBEDGraph () |
|  | |
| int | getDistanceTo (Annotation other) |
|  | |
| String | toCigar () |
|  | |
| List< Annotation > | intersect (List<?extends Annotation > others) |
|  | |
| int | compareTo (Annotation other) |
|  | |
| int | compareToAnnotation (Annotation b) |
|  | |
| int | compareToAnnotation (Annotation b, boolean useOrientation) |
|  | |
| boolean | equals (Annotation a) |
|  | |
| boolean | equals (Annotation a, boolean useOrientation) |
|  | |
| int | getSAMStart () |
|  | |
| int | getSAMEnd () |
|  | |

|  |  |
| --- | --- |
| Additional Inherited Members | |
| Static Public Member Functions inherited from umms.core.annotation.Gene | |
| static Collection< int[]> | findAllORFs (String sequence) |
|  | |
| static boolean | isStopCodon (String currentCodon) |
|  | |
| static Collection< Gene > | getOverlappers (Collection< Gene > genes, String chr, int start, int end, Strand strand, boolean ignoreOrientation) |
|  | |
| static int[] | findLongestORF (String sequence) |
|  | |
| Static Public Member Functions inherited from umms.core.annotation.BasicAnnotation | |
| static Annotation | createFromUCSC (String ucsc) |
|  | |
| static BasicAnnotation | fromFullInfoString (String fullInfoString) |
|  | |
| static void | main (String[] args) throws IOException |
|  | |
| Static Public Member Functions inherited from umms.core.annotation.AbstractAnnotation | |
| static Strand | getStrand (String orientation) |
|  | |
| static Strand | getStrand (char orientation) |
|  | |
| Static Public Attributes inherited from umms.core.annotation.Gene | |
| static final Pattern | START\_CODON\_PATRN = Pattern.compile("ATG", Pattern.CASE\_INSENSITIVE) |
|  | |
| static final String[] | STOP\_CODONS = {"TAG", "TAA", "TGA"} |
|  | |
| Static Public Attributes inherited from umms.core.annotation.AbstractAnnotation | |
| static final int | MAX\_DISTANCE = 1000000000 |
|  | |
| Protected Member Functions inherited from umms.core.annotation.Gene | |
| void | initFromGene (Gene gene) |
|  | |
| boolean | almostContainsRefSeq (Gene other, int exonFudgeFactor) |
|  | |
| Protected Attributes inherited from umms.core.annotation.BasicAnnotation | |
| CompoundInterval | blocks = new CompoundInterval() |
|  | |

## Constructor & Destructor Documentation

|  |  |  |  |  |  |
| --- | --- | --- | --- | --- | --- |
| umms.core.feature.GeneWindow.GeneWindow | ( | Gene | *gene* | ) |  |

Here is the caller graph for this function:

|  |  |  |  |  |  |
| --- | --- | --- | --- | --- | --- |
| umms.core.feature.GeneWindow.GeneWindow | ( | GeneWindow | *window* | ) |  |

|  |  |  |  |  |  |
| --- | --- | --- | --- | --- | --- |
| umms.core.feature.GeneWindow.GeneWindow | ( | Annotation | *window* | ) |  |

|  |  |  |  |  |  |
| --- | --- | --- | --- | --- | --- |
| umms.core.feature.GeneWindow.GeneWindow | ( | Collection<?extends Annotation > | *exons* | ) |  |

## Member Function Documentation

|  |  |  |  |  |  |
| --- | --- | --- | --- | --- | --- |
| void umms.core.feature.GeneWindow.addSourceAnnotation | ( | Annotation | *annotation* | ) |  |

Add a pointer to the annotation from which this Window originated

Parameters
:   |  |  |
    | --- | --- |
    | annotation |  |

Implements umms.core.feature.Window.

Here is the call graph for this function:

|  |  |  |  |  |  |
| --- | --- | --- | --- | --- | --- |
| int umms.core.feature.GeneWindow.compareTo | ( | Annotation | *g* | ) |  |

Here is the call graph for this function:

|  |  |  |  |  |  |
| --- | --- | --- | --- | --- | --- |
| boolean umms.core.feature.GeneWindow.equals | ( | Object | *o* | ) |  |

Here is the call graph for this function:

Here is the caller graph for this function:

|  |  |  |  |  |
| --- | --- | --- | --- | --- |
| int umms.core.feature.GeneWindow.getSize | ( |  | ) |  |

Returns the size of the window

Implements umms.core.annotation.Annotation.

Here is the caller graph for this function:

|  |  |  |  |  |
| --- | --- | --- | --- | --- |
| Collection<? extends Annotation> umms.core.feature.GeneWindow.getSourceAnnotations | ( |  | ) |  |

Returns
:   a collection of annotations from which this originated

Implements umms.core.feature.Window.

|  |  |  |  |
| --- | --- | --- | --- |
| Collection<Window> umms.core.feature.GeneWindow.getWindows | ( | int | *windowSize*, |
|  |  | int | *stepSize* |
|  | ) |  |  |

Get collection of windows spanning the annotation

Parameters
:   |  |  |
    | --- | --- |
    | windowSize | Window size |
    | stepSize | Step size |

Returns
:   The collection of windows

Implements umms.core.feature.Window.

Here is the call graph for this function:

---

The documentation for this class was generated from the following file:

- java/umms/core/feature/GeneWindow.java


---

Generated on Fri Aug 15 2014 15:11:10 for ESAT by  

 1.8.7
